# Mutation spectrum in a cohort with familial exudative vitreoretinopathy

**DOI:** 10.1002/mgg3.2021

**Published:** 2022-07-25

**Authors:** Ning Qu, Wei Li, Dong‐Ming Han, Jia‐Yu Gao, Zheng‐Tao Yang, Li Jiang, Tian‐Bin Liu, Yan‐Xian Chen, Xiao‐Sen Jiang, Liang Zhou, Ji‐Hong Wu, Xin Huang

**Affiliations:** ^1^ Guangdong and Shenzhen Key Laboratory of Reproductive Medicine and Genetics, The Center of Reproductive Medicine Peking University Shenzhen Hospital Shenzhen China; ^2^ College of Life Sciences University of Chinese Academy of Sciences Beijing China; ^3^ Department of Ophthalmology Laizhou City People's Hospital Yantai China; ^4^ Department of Ophthalmology Eye & ENT Hospital of Fudan University Shanghai China

**Keywords:** FEVR, genotype–phenotype analysis, mutation Spectrum, targeted sequencing

## Abstract

**Purpose:**

To expand the mutation spectrum of patients with familial exudative vitreoretinopathy (FEVR) disease.

**Participants:**

74 probands (53 families and 21 sporadic probands) with familial exudative vitreoretinopathy (FEVR) disease and their available family members (*n* = 188) were recruited for sequencing.

**Methods:**

Panel‐based targeted screening was performed on all subjects. Before sanger sequencing, variants of *LRP5*, *NDP*, *FZD4*, *TSPAN12*, *ZNF408*, *KIF11*, *RCBTB1*, *JAG1*, and *CTNNA1* genes were verified by a series of bioinformatics tools and genotype–phenotype co‐segregation analysis.

**Results:**

40.54% (30/74) of the probands were sighted to possess at least one etiological mutation of the nine FEVR‐causative genes. The etiological mutation detection rate was 37.74% (20/53) in family‐attainable probands while 47.62% (10/21) in sporadic cases. The diagnosis rate of patients in the early‐onset subgroup (≤5 years old, 45.4%) is higher than that of the children or adolescence‐onset subgroup (6–16 years old, 42.1%) and the late‐onset subgroup (≥17 years old, 39.4%). A total of 36 etiological mutations were identified in this study, comprising 26 novel mutations and 10 reported mutations. *LRP5* was the most prevalent mutant gene among the 36 mutation types with a percentage of 41.67% (15/36). Followed by *FZD4* (10/36, 27.78%), *TSPAN12* (5/36, 13.89%), *NDP* (4/36, 11.11%), *KIF11* (1/36, 2.78%), and *RCBTB1* (1/36, 2.78%). Among these mutations, 63.89% (23/36) were missense mutations, 25.00% (9/36) were frameshift mutations, 5.56% (2/36) were splicing mutations, 5.56% (2/36) were nonsense mutations. Moreover, the clinical pathogenicity of these variants was defined according to American College of Medical Genetics (ACMG) and genomics guidelines: 41.67% (15/36) were likely pathogenic variants, 27.78% (10/36) pathogenic variants, 30.55% (11/36) variants of uncertain significance. No etiological mutations discovered in the *ZNF408*, *JAG1,* and *CTNNA1* genes in this FEVR cohort.

**Conclusions:**

We systematically screened nine FEVR disease‐associated genes in a cohort of 74 Chinese probands with FEVR disease. With a detection rate of 40.54%, 36 etiological mutations of six genes were authenticated in 30 probands, including 26 novel mutations and 10 reported mutations. The most prevalent mutated gene is *LRP5*, followed by *FZD4*, *TSPAN12*, *NDP*, *KIF11*, and *RCBTB1*. In total, a de novo mutation was confirmed. Our study significantly clarified the mutation spectrum of variants bounded up to FEVR disease.

## Graphical abstract

Our genetic finding can serve as an efficient reference and comprehensive research strategy for the design of panel‐based genetic diagnostic testing, genetic counseling, and future diagnosis for suspected patients with family exudative vitreoretinopathy disease.

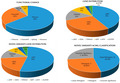

## 1 INTRODUCTION

Familial exudative vitreoretinopathy (FEVR, OMIM 133780) is a rare genetic disorder which is characterized by deviant development of peripheral retinal vessels (Criswick & Schepens, 1969; Poulter et al., 2016; van Nouhuys, 1989). Criswick and Schepens firstly described this disease in 1969 (Criswick & Schepens, 1969). The clinical manifestations are widely variable in diverse FEVR patients, ranging from asymptomatic peripheral vascular abnormalities to congenital blindness (van Nouhuys, 1989). The sight‐threatening features of the FEVR phenotype are considered secondary to retinal avascularity and develop because of the resulting retinal ischemia; they include the development of hyperpermeable blood vessels, neovascularization, vitreoretinal traction, retinal folds, and retinal detachments (Poulter et al., 2016). Based on the diverse clinical patterns (including the presence avascular zone in the extreme periphery, retinal detachment, neovascularization, macular ectopia, falciform retinal fold, vitreoretinal adhesion, and exudative retinal detachment) in FEVR patients, a grading system was proposed to divide FEVR disorder into five types (Miyakubo et al., 1984). FEVR can be inherited in patterns of autosomal dominant pattern, autosomal recessive, and X‐linkage inheritance, and the most prevalent pattern of inheritance is autosomal dominant (Chen et al., 1993; Laqua, 1980; Rao et al., 2017).

Specific variants of *LRP5* (OMIM 603506), *TSPAN12* (OMIM 613138), *ZNF408* (OMIM, 616454), *NDP* (OMIM 300658), *FZD4* (OMIM 604579), and *KIF11* (OMIM, 148760) were known to be responsible for FEVR (Collin et al., 2013; Liu et al., 2017; Nikopoulos et al., 2010; Robitaille et al., 2002, 2014; Royer et al., 2003). *NDP*, *FZD4*, *LRP5*, and *TSPAN12* were genes involved in Wnt/Norrin signaling pathway, which significantly affects the development of ocular and retinal vasculature. Moreover, previous reports had revealed that *RCBTB1* (OMIM 607867) gene is also associated with FEVR (Wu et al., 2016). Furthermore, the latest study elucidated that notch ligand *JAG1* gene (OMIM 601920) might be a novel candidate gene for FEVR via knockout mouse model and related analysis (Zhang et al., 2020). In addition, a recent study reported that three heterozygous mutations (p.F72S, p.R376Cfs*27, and p.P893L) in α‐catenin (*CTNNA1*, OMIM 608970) cause FEVR by overactivating the Norrin/β‐catenin signaling pathway and disrupting cell adherens junctions (Zhu et al., 2021). In fact, previous studies have depicted the partial mutation spectrum of *LRP5*, *NDP*, *FZD4*, *TSPAN12*, *ZNF408*, *KIF11*, *RCBTB1*, and *JAG1* gene (Li et al., 2018; Tang et al., 2017; Wu et al., 2016; Zhang et al., 2020). Nonetheless, variants in these genes can explain only less than half of the FEVR patients. Few studies have portrayed the overall mutation spectrum of all nine genes. A more comprehensive landscape needs to be delineated, so thence we can better understand the genetic mechanism of FEVR and its benefits to clinical practice.

In this study, we systematically screened nine reported FEVR disease‐associated genes (*LRP5*, *FZD4*, *TSPAN12*, *NDP*, *KIF11*, *ZNF408*, *RCBTB1*, *JAG1*, and *CTNNA1*) in a cohort of 74 Chinese probands and their available family members. The diagnosis of the FEVR patients was performed by professional ophthalmologists. We amplified the mutation spectrum of patients with FEVR disease which can not only benefit clinical practice but also assist in designing the ophthalmic panel.

## 2 MATERIALS AND METHODS

### 2.1 Subjects collection and clinical assessment

This study was approved by the Ethics Committee of the Eye & ENT Hospital of Fudan University and complies with the tenets of the Declaration of Helsinki for medical research related to human subjects. All participants had signed the written informed consent. For minors, obtain the consent from the legal guardian. 74 probands and their available family members (total participants: 188) who came to Eye & ENT Hospital of Fudan University in 2017–2020 were recruited for this study. Encompassing ophthalmologic examination was performed on all subjects, including the best corrected visual acuity (BCVA), slit‐lamp biomicroscopy, color vision testing, intraocular pressure, Humphrey perimetry, electroretinography, fundus autofluorescence, and spectral domain optical coherence tomography (ST‐OCT). The final clinical diagnosis of FEVR patients was appraised by professional ophthalmologists. All these FEVR probands are divided into three groups according to the age of onset, including the early‐onset subgroup (≤5 years, *n* = 22), the children or adolescence subgroup (6–16 years, *n* = 19), and the late‐onset subgroup (≥17 years, *n* = 33). The details of this FEVR cohort are depicted in Table 1.

**TABLE 1:** Summary of clinical details of enrolled probands

Sample category	Female/male	Diagnostic yield
Total FEVR probands	32/42	40.54% (30/74)
Famillies	20/33	37.74% (20/53)
Sporadic	12/9	47.62% (10/21)
Age of onset (yrs)		
≤5	10/12	45.4% (10/22)
6–16	7/12	42.1% (8/19)
≥17	15/18	39.4% (13/33)

### 2.2 DNA sample collection

Peripheral blood samples were collected from all subjects. We used the Flexigene DNA Kit to exact genomic DNA. Collected DNA samples were stored at −20°C.

### 2.3 Targeted exome sequencing

Panel‐based next‐generation sequencing was accomplished on all subjects recruited in this study. We designed a high‐throughput chip that contains 792 eye‐diseases‐related genes (Supplementary Table S1) to actualize precise targeted sequencing. The Agilent SureSelect Target Enrichment Kit (Agilent Technologies, Inc., USA) was utilized to conduct DNA libraries and then the reads were sequenced on MGISEQ‐2000 platform (DNBSEQ‐G400) (Gao et al., 2019; Li et al., 2021). The size of the generated reads during paired‐end sequencing was 100 bp and the average depth was 704X while the median depth was 677X.

### 2.4 Genetic analysis

Sequence reads were mapped to the reference human genome (hg38) using Burrows‐Wheeler aligner version 0.7.10 (BWA‐ MEM, http://bio‐bwa.sourceforge.net/) (Li & Durbin, 2009). CNVkit (v0.8.5) was used to analyze the sequencing coverage and copy number in the aligned sequencing reads of targeted DNA sequencing data (Eric et al., 2016; Yao et al., 2019). All obtained variants experienced an annotation of 1000 Genomes Project (http://browser.1000genomes.org/), the Single Nucleotide Polymorphism Database (http://www.ncbi.nlm.nih.gov/projects/SNP), Exome Sequencing Project v.6500 (http://evs.gs.washington.edu/EVS/), Exome Aggregation Consortium (http://exac.broadinstitute.org) database and the Genome Aggregation Database (gnomAD, https://gnomad.broadinstitute.org). We used 0.01% as the threshold value of minor allele frequency (MAF) to select variants for further research. Variants with a MAF greater than the threshold value were discarded. Moreover, bioinformatics tools including Sorting Intolerant from Tolerant (SIFT, https://sift.bii.a‐star.edu.sg/), Likelihood Ratio Test Query (LRT, http://www.genetics.wustl.edu/jflab/lrt_query.html), FATHMM (http://fathmm.biocompute.org.uk/), and MutationTaster (http://www. mutationtaster.org/) were utilized to predict the deleteriousness of variants. Furthermore, genotype–phenotype co‐segregation analysis and sanger sequencing were adopted to verify these variants. ACMG genomics standards and guidelines were used to classify the pathogenicity of variants. In accordance with ACMG genomics standards and guidelines, We utilized two sets of criteria: one for classification of Pathogenic or Likely Pathogenic variants, and one for classification of Benign or Likely Benign variants (Richards et al., 2015). Each pathogenic criterion is weighted as very strong (PVS1), strong (PS1–4); moderate (PM1–6), or supporting (PP1–5) and each benign criterion is weighted as stand‐alone (BA1), strong (BS1–4) or supporting (BP1–6). Based on the concepts and meaning of each criterion described in the ACMG genomics standards and guidelines, We carefully checked the criterions our variants meet via VarSome (https://varsome.com/) and our manual proofing. Finally, we determined the pathogenicity of our variants via the rules for combining criteria to classify Sequence variants claimed in the ACMG genomics standards and guidelines.

### 2.5 Statistical analysis

We calculated the detection yield and clarified the mutation spectrum in both the family‐attainable cohort and the sporadic cohort. The pattern of inheritance in families was investigated and described in the “Results” section. We also depicted the spectrum in subjects with novel variants and reported variants. Novel mutation was defined if it had not been reported in previous literature. De novo mutation was ascertained if it did not appear in the biological parents of the mutation‐carried patients. The paternity would be checked when we reported a de novo variant.

## 3 RESULTS

### 3.1 General spectrum of this cohort

The detection yield in the whole FEVR cohort is 40.54% (30/74). Among them, 23 subjects carried one mutation, 6 subjects carried 2 mutations and 1 subject carried 3 mutations. 66.67% (20/30) of these etiological mutations carried cases have available family members and 39.39% (10/30) are isolated cases. The detection rate is different in two groups: 37.74% (20/53) in family‐attainable probands while 47.62% (10/21) in sporadic cases.

**FIGURE 1:**
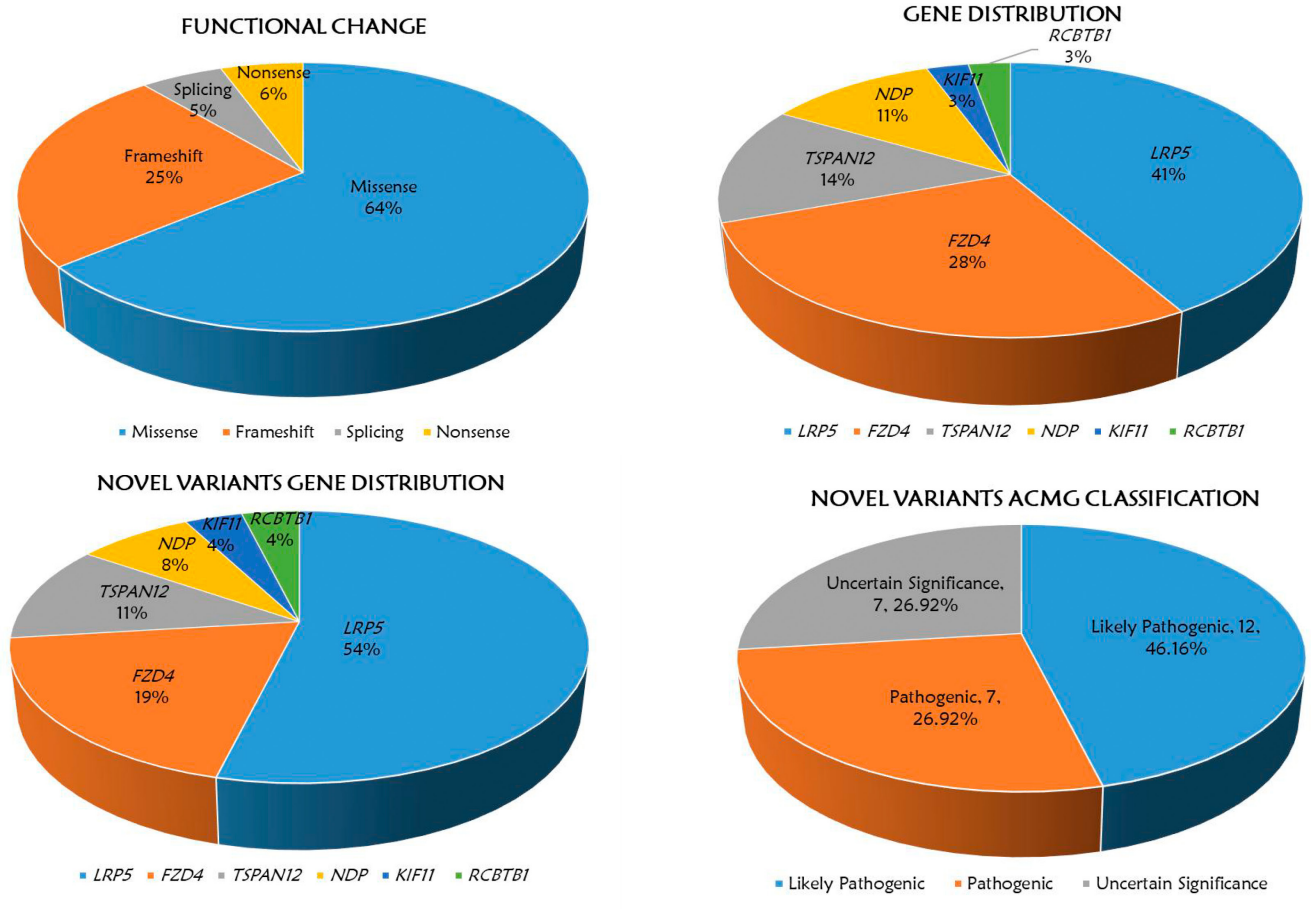
(a) functional change of 36 variants in the whole FEVR cohort. (b) Gene distribution of 36 variants of the 6 FEVR‐causative genes. (c) Novel variants gene distribution of 26 novel variants of the 6 FEVR‐causative genes. (d) novel variants ACMG significance of the 26 novel variants.

**FIGURE 2:**
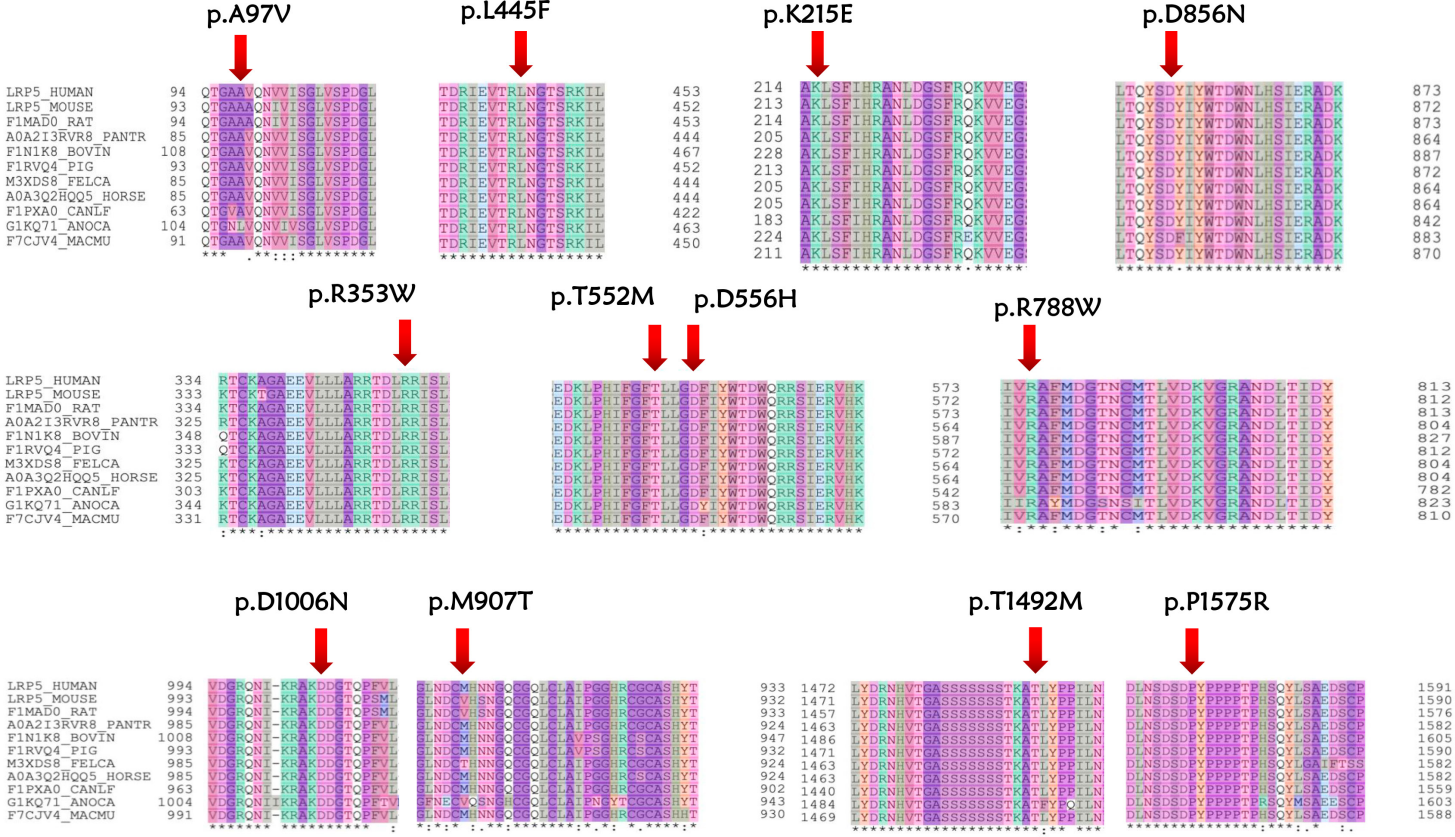
Multiple sequence alignment of different species of the 12 missense variants of *LRP5* gene. (the red arrow represents mutation sites). The 11 species are shown below: HUMAN, Homo sapiens; MOUSE, Mus musculus; RAT, Rattus norvegicus; PANTR, Pan troglodytes; BOVIN, Bos Taurus; PIG, Sus scrofa; FELCA, Felis catus; HORSE, Equus caballus; CANLF, Canis lupus familiaris; ANOCA, Anolis carolinensis; MACMU, Macaca mulatta.

36 mutations of *LRP5*, *FZD4*, *TSPAN12*, *NDP*, *KIF11*, and *RCBTB1* genes were confirmed in the entire FEVR group, including 26 novel mutations and 10 reported mutations (Supplementary Table S2). No evident discovery was uncovered in *JAG1* and *ZNF408* gene in this whole FEVR cohort. Among these mutations, 63.89% (23/36) were missense mutations, 25.00% (9/36) were frameshift mutations, 5.56% (2/36) were splicing mutations and 5.56% (2/36) were nonsense mutations (Figure 1a). Additionally, as depicted in Figure 1b, *LRP5* gene made up the largest proportion in the 36 types of mutations with a percentage of 41.67% (15/36) and followed by *FZD4* (10/36, 27.78%), *TSPAN12* (5/36, 13.89%), *NDP* (4/36, 11.11%), *KIF11* (1/36, 2.78%), and *RCBTB1* (1/36, 2.78%). Among the 15 mutations of *LRP5* gene, missense accounts for the largest proportion with a percentage of 80.0% (12/15). Multiple sequence alignment of different species of the 12 missense mutations is shown in Figure 2. The most prevalent mutations were two missense mutations of *TSPAN12* gene (c.194C>T, p.P65L) and *LRP5* gene (c.518C>T, p.T173M), both of them were detected in two FEVR probands. Moreover, the clinical pathogenicity of these mutations was defined according to ACMG and genomics guidelines: 41.67% (15/36) likely pathogenic mutations, 27.78% (10/36) pathogenic mutations, 30.55% (11/36) variants of uncertain significance.

### 3.2 Spectrum of variants in family‐accessible subjects

71.62% (53/74) of the FEVR probands have available family members. We firstly investigated the mutation spectrum of variants in family‐attainable FEVR probands. 37.74% (20/53) of the probands harbored at least one mutant allele of the six genes above. 75.0% (15/20) of the mutation‐carried subjects carried one variant, 20.0% (4/20) of the patients were found to harbor two variants and 5.00% (1/20) of the patients harbor three variants. The pedigrees of family‐attainable probands are depicted in supplementary Figure S1. A total of 24 mutations were ascertained in these families (Table 2), including *FZD4* (29.17%, 7/24), *KIF11* (4.17%, 1/24), *LRP5* (33.33%%, 8/24), *NDP* (12.50%, 3/24), *RCBTB1* (4.17%, 1/24), *TSPAN12* (16.67%, 4/24). 70.83% (17/24) were novel variants while 29.17% (7/24) variants have been reported in the previous literature. Co‐segregation analysis in family members was performed in all 24 variants. One missense mutation of *TSPAN12* (c.194C>T, p.P65L) gene appeared twice in these families. The final pathogenicity was determined according to ACMG and genomics guidelines. 37.5% (9/24) were likely pathogenic mutations while 20.83% (5/24) were pathogenic mutations. 41.67% (10/24) were variants of uncertain significance. The 24 mutations detected in the 20 families included missense (19/24, 79.17%), splicing (1/24, 4.17%), and frameshift (4/24, 16.67).

**TABLE 2:** 24 variants detected in family‐attainable probands

Num	Gene	Nucleotide change	Amino acid change	Functional change	In silico prediction	ACMG category	Patients ID	If reported
1	*TSPAN12*	c.194C>T	p.Pro65Leu	Missense	T,D,D,T	VUS	F1,F10	Tang et al. (2017)
2	*FZD4*	c.892A>G	p.Lys298Glu	Missense	T,D,D,T	VUS	F2	Novel
3	*FZD4*	c.893delA	p.Lys298Argfs7	Frameshift	–,–,–,–	LP	F2	Novel
4	*FZD4*	c.896_898delACA	p.Asn299del	InframeDeletion	–,–,–,–	VUS	F2	Novel
5	*LRP5*	c.1666G>C	p.Asp556His	Missense	D,U,D,D	LP	F3	Novel
6	*LRP5*	c.1655C>T	p.Thr552Met	Missense	D,U,D,D	VUS	F4	Narumi et al. (2010)
7	*LRP5*	c.4724C>G	p.Pro1575Arg	Missense	D,U,D,D	LP	F4	Novel
8	*LRP5*	c.3016G>A	p.Asp1006Asn	Missense	T,U,D,D	VUS	F5	Novel
9	*NDP*	c.137A>G	p.Asp46Gly	Missense	D,D,D,D	P	F6	Novel
10	*TSPAN12*	c.146C>T	p.Thr49Met	SpliceSite	D,D,D,T	VUS	F7	Yang et al. (2011)
11	*RCBTB1*	c.1238T>G	p.Ile413Ser	Missense	T,D,D,T	LP	F8	Novel
12	*LRP5*	c.1333C>T	p.Leu445Phe	Missense	D,U,D,D	LP	F9	Novel
13	*KIF11*	c.2220_2221del	p.Cys740*fs1	Frameshift	–,–,–,–	LP	F11	Novel
14	*TSPAN12*	c.476G>A	p.Cys159Tyr	Missense	D,D,D,D	VUS	F12	Novel
15	*LRP5*	c.2720T>C	p.Met907Thr	Missense	T,N,P,D	VUS	F13	Novel
16	*NDP*	c.343C>G	p.Arg115Gly	Missense	T,D,D,D	P	F13	Novel
17	*LRP5*	c.290C>T	p.Ala97Val	Missense	T,N,P,D	VUS	F14	Novel
18	*NDP*	c.338G>A	p.Gly113Asp	Missense	D,D,D,D	P	F15	Musada et al. (2016)
19	*LRP5*	c.4475C>T	p.Thr1492Met	Missense	D,U,D,D	LP	F16	Novel
20	*FZD4*	c.1589G>A	p.Gly530Glu	Missense	D,D,D,T	LP	F17	Li et al. (2018)
21	*FZD4*	c.1188_1192delTACTT	p.Phe396Leufs*61	Frameshift	–,–,–,–	P	F18	Novel
22	*FZD4*	c.205C>T	p.His69Tyr	Missense	D,D,D,D	VUS	F18,F19	Seo et al. (2015)
23	*FZD4*	c.313A>G	p.Met105Val	Missense	T,D,D,T	LP	F19	Tang et al. (2017)
24	*TSPAN12*	c.1A>G	p.Met1Val	Missense	D,D,D,T	P	F20	Novel

*Notes*: (a) D, damaging; T, tolerant; N, Neutral; and P, polymorphism. In silico prediction was performed by SIFT, LRT, Mutation Taster, and FATHMM. (b) P, Pathogenic; LP, Likely Pathogenic; VUS, Uncertain Significance; LB, Likely Benign; and B, Benign. (c) F, Family; S, Sporadic.

Of all the 24 mutations detected in the families, 12.50% (3/24) were inherited in the patterns of X‐linkage recessive inheritance. Including three missense mutations (c.338G>A, c.137A>G, c.343C>G) of *NDP* gene. One de novo mutation (*LRP5*, c.4724C>G, p.P1575R) was uncovered in family 4 (F4). The remaining 20 mutations were inherited in autosomal dominant pattern. Among them, 10.0% (2/20) were considered as pathogenic mutations according to the methods we described in Materials and Methods, 40.0% (8/20) were determined to be likely pathogenic mutations and 50.0% (10/20) were thought to be variants of uncertain significance. No variants were sighted to be inherited in autosomal recessive pattern.

**FIGURE 3:**
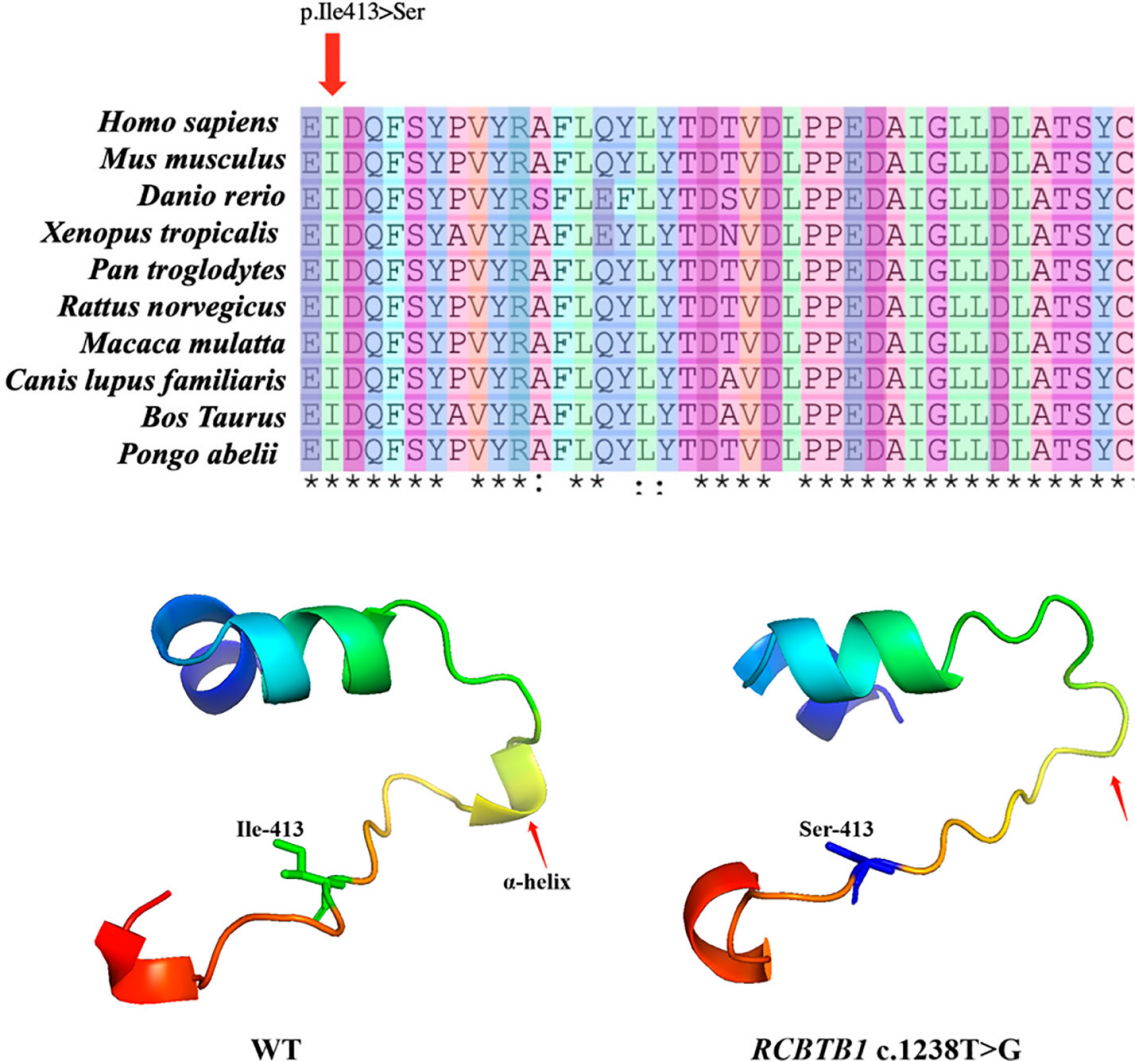
(a) multiple sequence alignment of different species of the mutation (the red arrow represents mutation sites). (b) 3D structural model of the wild type (WT) and mutant residues (*RCBTB1* c.1238T>G). The red arrow represents the α‐helix change of the peptide chain structure.

One novel missense mutation (c.1238T>G, p.I413S) of *RCBTB1* gene was found in two members of family 8 (F8), the proband and his affected biological father. This mutation is located in a highly evolutionarily conserved region (Figure 3a) and altered the corresponding amino acid from isoleucine to serine. The 3D structural model of these amino changes is portrayed in Figure 3b. Using PyMOL (https://pymol.org/2/) to visualize the structure of peptide chain, and the results show that the α‐helix of mutant protein is missing (Janson & Paiardini, 2020). No variants of the other eight genes were checked out in the two FEVR subjects. This variant was predicted to be likely pathogenic in accordance with ACMG and genomics guideline, hence we considered it as a candidate causative factor for the FEVR cases in this family.

### 3.3 Spectrum of variants in sporadic FEVR cases

21 probands with FEVR disease did not have accessible family members in this study and 12 mutations were confirmed in 10 (47.62%, 10/21) isolated cases (Table 3). 75.0% (9/12) were novel variants while 25.0% (3/12) were reported variants. 80.0% (8/10) of these sporadic cases harbored one variant while 20.0% (2/10) carried two variants. Of all the 12 variants, 33.33% (4/12) were missense mutations, 41.67% (5/12) were frameshift mutations, 8.3% (1/12) were splicing mutations and 16.67% (2/12) were nonsense mutations. 50.0% (6/12) of the variants were conceived as likely pathogenic, 41.67% (5/12) were considered as pathogenic, 8.33% (1/12) were thought to be variant of unknown significance.

**TABLE 3:** 12 variants detected in sporadic probands

Num	Gene	Nucleotide change	Amino acid change	Functional change	In silico prediction	ACMG category	Patients ID	If reported
1	*FZD4*	c.1282_1285delGACA	p.Asp428Serfs2	Frameshift	–,–,–,–	P	S1	Seo et al. (2015)
2	*FZD4*	c.1516A>T	p.Lys506*	Nonsense	–,D,D,–	P	S2	Novel
3	*FZD4*	c.40_49delCCCGGGGGCG	p.Pro14Serfs*44	Frameshift	–,–,–,–	LP	S3	Khan et al. (2017)
4	*LRP5*	c.2362C>T	p.Arg788Trp	Missense	D,U,D,D	VUS	S4	Novel
5	*LRP5*	c.1057C>T	p.Arg353Trp	Missense	D,U,D,D	LP	S5	Novel
6	*LRP5*	c.1801G>A	p.Gly601Arg	SpliceSite	D,U,D,D	LP	S5	Novel
7	*LRP5*	c.2013delC	p.Thr672Argfs*25	Frameshift	–,–,–,–	P	S6	Novel
8	*LRP5*	c.643A>G	p.Lys215Glu	Missense	D,U,D,D	LP	S6	Novel
9	*LRP5*	c.2566G>A	p.Asp856Asn	Missense	D,U,D,D	LP	S7	Novel
10	*LRP5*	c.4447_4448insC	_	Frameshift	–,–,–,–	LP	S8	Novel
11	*NDP*	c.268delC	p.Arg90Valfs*14	Frameshift	–,–,–,–	P	S9	Seo et al. (2015)
12	*TSPAN12*	c.352G>T	p.Glu118Ter	Nonsense	–,D,D,–	P	S10	Novel

*Notes*: (a) D, damaging; T, tolerant; N, Neutral; and P, polymorphism. In silico prediction was performed by SIFT, LRT, Mutation Taster, and FATHMM. (b) P, Pathogenic; LP, Likely Pathogenic; VUS, Uncertain Significance; LB, Likely Benign; and B, Benign. (c) F, Family; S, Sporadic.

### 3.4 Spectrum of FEVR subjects with novel variants

26 novel variants involved in 21 FEVR cases were detected in this study totally. 92.31% (24/26) of these were heterozygous and 7.69% (2/26) were hemizygous cases. As shown in Figure 1c, of the 26 novel variants, *LRP5* gene occupied the largest proportion with a percentage of 53.85% (14/26) and followed by *FZD4* (5/26, 19.23%), *TSPAN12* (3/26, 11.54%), *NDP* (2/26, 7.70%), *KIF11* (1/26, 3.85%), and *RCBTB1* (1/26, 3.85%). 46.16% (12/26) of the variants were considered as likely pathogenic mutations, 26.92% (7/26) were defined as pathogenic mutations, 26.92% (7/26) were thought to be variants of uncertain significance (Figure 1d).

Missense mutations accounted for the largest proportion of all the 26 variants with a percentage of 65.38% (17/26), followed by frameshift mutations (23.10%, 6/26), nonsense mutations (7.70%, 2/26), and splicing mutations (3.85%, 1/26).

### 3.5 Spectrum of FEVR subjects with reported variants

10 reported variants were ascertained in this study in total (Supplementary Table S2). Different from the novel variants, *FZD4* gene accounted for the largest proportion of these reported variants with a percentage of 50.0% (5/10). The following was *TSPAN12* gene (2/10, 20.0%), *NDP* gene (2/10, 20.0%), and *LRP5* gene (1/10, 10.0%). The most prevalent mutation type was missense mutation (6/10, 60.0%), frameshift mutation (3/10, 30.0%), and splicing mutation (1/10, 10.0%). Based on the methods we stated in “Materials and methods” section, 30.0% (3/10) of the reported variants were considered as likely pathogenic variants, 30.0% (3/10) were defined as pathogenic variants, 40.0% (4/10) were thought to be variants of uncertain significance.

## 4 DISCUSSION

FEVR is a rare inherited disease that significantly impairs the vision of affected patients (Finis et al., 2010; Gilmour, 2015; Tauqeer & Yonekawa, 2018). The partial spectrum of these genes has been represented in the previous literature (Li et al., 2018; Lin et al., 2010; Rao et al., 2017). Rao et al. (2017) screened six known disease‐causing genes (*LRP5*, *KIF11*, *NDP*, *ZNF408*, *FZD4*, *TSPAN12*) in 31 pedigrees with FEVR and depicted the mutation spectrum in Chinese (Rao et al., 2017). Li et al. (2018) represented the spectrum of variants in 389 Chinese probands with familial exudative vitreoretinopathy (Li et al., 2018). But few overall spectrum of these nine genes has been presented.

We depict the comprehensive mutation spectrum of nine FEVR‐causative genes and the detection yield is 40.54% (30/74) in FEVR cases. We uncover 26 novel variants of the six genes in total and 10 reported mutations. In the light of ACMG and genomics guidelines, 41.67% (15/36) of these variants were considered as likely pathogenic variants, 27.78% (10/36) were pathogenic variants, 30.55% (11/36) variants were defined as variants of uncertain significance. In correspondence with the previous reports, *LRP5* accounts for the largest percentage of all the mutant genes in our study (Li et al., 2018; Tang et al., 2017). We divide the cohort into two groups: the probands with available family members and isolated cases. Different detection rates were revealed in these two groups: 37.74% (20/53) in family‐attainable probands while 47.62% (10/21) in sporadic cases. 24 variants were detected in the family‐attainable probands. Among them, 12.50% (3/24) inherited in the patterns of X‐linkage recessive inheritance. One de novo mutation (*LRP5*, c.4724C>G, p.P1575R) was uncovered in family 4 (F4). The paternity in this family was checked seriously. The remaining 20 (83.33%, 20/24) variants were inherited in autosomal dominant pattern and no variants were sighted to be inherited in autosomal recessive pattern. Comparing the detection rate of FEVR probands in the three age groups, there was no significant difference between the early‐onset subgroup, the child or adolescent subgroup, and the late‐onset subgroup, indicating that genetic factors are the main cause of FEVR patients at all stages.

A series of 389 consecutive FEVR patients from 389 families were sequenced for *FZD4*, *LRP5*, *NDP*, *TSPAN12*, *ZNF408*, and *KIF11* genes. A total of 101 potentially pathogenic variants (PPV) and 49 variants of uncertain significance (VUS) were identified. Of them, 110 probands carried PPV (28.3%), and 51 probands carried VUS (13.1%). PPV in *FZD4*, *LRP5*, *TSPAN12*, *NDP*, *ZNF408*, and *KIF11* accounted for 8.48%, 9.00%, 5.91%, 4.63%, 0.77%, and 0.77%, respectively (Li et al., 2018). Another study recruited 100 probands and their families for genetic screening of *LRP5*, *NDP* and *TSPAN12* and identified 23 pathogenic mutations in 23 unrelated probands (10/23 in *LRP5*, 8/23 in *TSPAN12*, and 5/23 in *NDP*). The overall detection rate of the three known genes is 23%. The mutations of *LRP5* and *TSPAN12* are more frequent, accounting for 10% and 8%, respectively (Tang et al., 2017). The overall detection rate of the nine known genes in our study is 40.54% (30/74), and the high‐frequency detection genes are *LRP5* (41.67%), *FZD4* (27.78%), *TSPAN12* (13.89%), *NDP* (11.11%), *KIF11* (2.78%), and *RCBTB1* (2.78%).

Vitreoretinopathy is a group of genetic and clinically heterogeneous disorders in retinal vascular development. Recently, novel etiological genes have been discussed and reported. Two heterozygous frameshift mutations in the *RCBTB1* gene were identified in three Taiwanese cases, and it was also proved that the *RCBTB1* gene has a haploinsufficient mechanism, involved in the Norrin/FZD4 signaling pathway, and the transgenic fli1:EGFP zebrafish with rcbtb1 knockdown exhibited abnormalies in intraocular blood vessels (Wu et al., 2016). However, another refutation study identified biallelic mutations in *RCBTB1* in an isolated patient with retinitis pigmentosa (RP, MIM 268000), and heterozygous truncated variants were distributed in different eye disease phenotypes but were not enriched in FEVR, emphasizing that it was not related to specific eye disease phenotypes (Yang et al., 2021). In our study, a missense mutation in the *RCBTB1* gene was identified in the proband and the affected father of family 8 (F8), and multiple public databases and function prediction software were used for deleterious assessment. No other known mutations in FEVR‐related genes have been detected, and the putative genetic cause of vitreoretinopathy phenotype is caused by the *RCBTB1* gene. FEVR is a genetic and clinically heterogeneous disease. A reasonable explanation is that the *RCBTB1* gene may cause the RP phenotype in the recessive mode, and the phenotype of vitreoretinopathy in dominant mode, sometimes as a syndrome or asymptomatic phenotype. Furthermore, the latest study elucidated that notch ligand *JAG1* might be a novel candidate gene for FEVR (Zhang et al., 2020). This gene was also included in our panel and we screened this gene in all the subjects. However, no reliable pathogenic variants were founded in patients with FEVR disease. Besides, CNVkit was used to trace DNA copy number mutation in the whole cohort but no reliable result was discovered. 44 probands were negative for our 792 ophthalmic‐disease‐related genes‐based panel sequencing. This phenomenon indicated that novel genes may be responsible for FEVR disease and the pathogenicity of *JAG1* gene to FEVR disease need to be further investigated. A study of *CTNNA1* identified three heterozygous mutations, demonstrating that FEVR‐related mutations lead to excessive activation of Norrin/β‐catenin signaling. After evaluating *CTNNA1* mutations in the FEVR population, no potential pathogenic mutations have been found in our FEVR cohort (Zhu et al., 2021). Another CTNNB1 was reported to cause FEVR, but it was not included in our detection panel list (Dixon et al., 2016; Rossetti et al., 2021). In the future, we will further improve and update the range of genes covered by our panel.

In the study of 74 subjects from 17 separate families (including 17 patients and 57 family members), 43% of FEVR patients had detectable disease‐causative mutations. However, only 8% of cohort patients reported a positive family history of FEVR in first‐degree relatives. However, among asymptomatic family members, 58% of clinical or angiographic results are consistent with stage 1 or 2 FEVR, and 21% of clinical or angiographic results are consistent with stage 3, 4, or 5 FEVR (Kashani et al., 2014). Although the genetic pattern of FEVR is known, previous studies have not attempted to systematically recruit asymptomatic family members of FEVR patients for clinical and angiographic examinations and may underestimate the occurrence of the disease (albeit asymptomatic) (Pendergast & Trese, 1998; Ranchod et al., 2011). Obviously, clinical examination alone is not enough to identify the microvascular changes in early FEVR, and it is difficult to identify the subtle peripheral findings without angiography or insufficient angiography. The clinical significance of identifying patients with early and asymptomatic FEVR is not trivial. The identification of asymptomatic family members in children of childbearing age is important for potential genetic testing, newborn screening for future children, or both.

In conclusion, we significantly expand the mutation spectrum of patients with FEVR disease. Our study might cover the most FEVR‐associated genes. Our comprehensive landscape can assist in understanding the genetic mechanism of FEVR and benefit clinical practice. With a detection yield of 40.54% (30/74), a total of 36 variants were identified after rigorous filtering, including one de novo mutation. We investigated the different mutation spectrum in the family‐attainable cohort and the sporadic cases. Also, we ascertained the different patterns of inheritance of FEVR patients, including autosomal dominant pattern and X‐linkage recessive inheritance. But no DNA copy number mutation were identified in the whole FEVR cohort. Besides, no reliable variants of *JAG1* and *CTNNA1* genes were detected in all the FEVR patients. Based on these results, further research about the genotype and phenotype relationship is needed to be invested in the future therefore we can better understand the mechanism of FEVR disease.

### AUTHOR CONTRIBUTION

*Conceptualization*: Ning Qu, Wei Li, Liang Zhou, Ji‐Hong Wu, and Xin Huang. *Data curation*: Wei Li, Dong‐Ming Han, Jia‐Yu Gao, Zheng‐Tao Yang, Li Jiang, Tian‐Bin‐Liu, and Xiao‐Sen Jiang. *Formal analysis*: Wei Li, Dong‐Ming Han, Jia‐Yu Gao, and Zheng‐Tao Yang. *Funding acquisition*: Ning Qu, Liang Zhou, Yan‐Xian Chen, Ji‐Hong Wu, and Xin Huang. *Investigation*: Wei Li, Ning Qu, and Ji‐Hong Wu. *Methodology*: Ning Qu and Wei Li. *Project administration*: Ning Qu and Wei Li. *Resources*: Wei Li, Ning Qu, Li Jiang, Liang Zhou, Ji‐Hong Wu, and Xin Huang. *Supervision*: Xin Huang, Liang Zhou, and Ji‐Hong Wu. *Visualization*: Wei Li. *Writing‐original draft, review, and editing*: Ning Qu, Wei Li, Liang Zhou, Ji‐Hong Wu, and Xin Huang.

### FUNDING INFORMATION

This work was supported by the foundation of Clinical Research of Shenzhen Municipal Health Commission (SZLY2017006), the foundation of Research Project of Shenzhen Municipal Health System (SZXJ2018075). Sponsored by Program of Shanghai Academic Research Leader (20XD1401100), Program for Outstanding Medical Academic Leader (2019LJ01), Supported by NSFC 81770925, 81790641, 82070975, Shanghai Municipal Science and Technology Major Projects (2018SHZDZX05).

### CONFLICT OF INTEREST

The authors declare that the research was conducted in the absence of any commercial or financial relationships that could be construed as a potential conflict of interest.

## Supporting information


Figure S1

Table S1

Table S2
