# Supplementary material for: Mutation spectrum in a cohort with familial exudative vitreoretinopathy
Source: Mol Genet Genomic Med. 2022 Jul 25;10(9):e2021. doi: 10.1002/mgg3.2021 (PMC9482396; doi:10.1002/mgg3.2021)

Supplementary Figure 1. Pedigrees of 20 variant-carried families. Filled symbol means affected patients with FEVR, the unfilled symbol indicates unaffected individuals, and the dotted symbols denote carriers. "M" indicates mutant alleles, "Y" indicates Y chromosome normal alleles, "+" indicates normal allele, and "*" indicates de novo

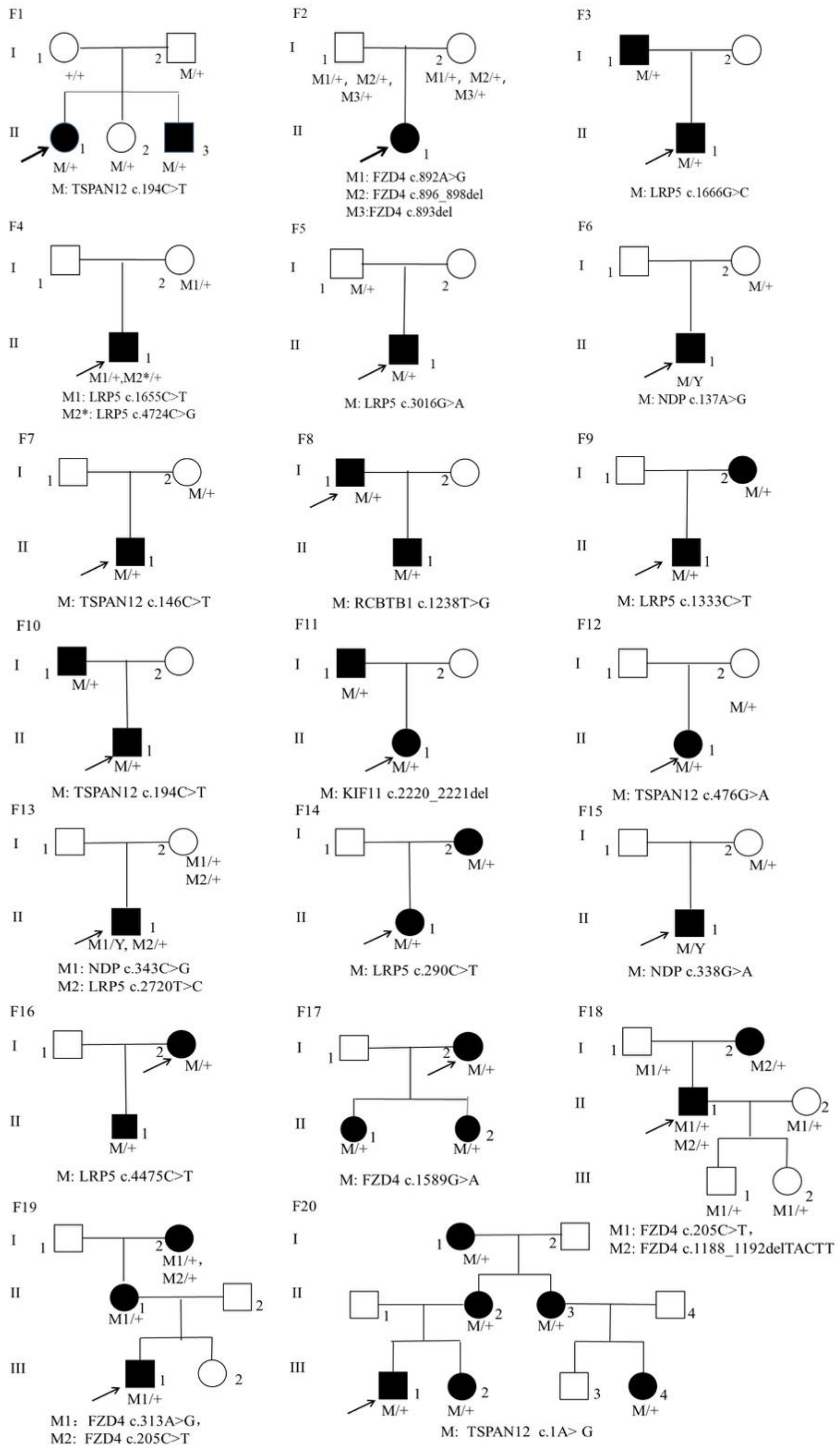

Supplement: Supplementary file 1 — Figure S1 [file MGG3-10-e2021-s002.pdf]
