# Supplementary material for: Mutation spectrum in a cohort with familial exudative vitreoretinopathy
Source: Mol Genet Genomic Med. 2022 Jul 25;10(9):e2021. doi: 10.1002/mgg3.2021 (PMC9482396; doi:10.1002/mgg3.2021)
Supplement: Supplementary file 2 — Table S1 [file MGG3-10-e2021-s001.pdf]

Supplementary Table 1. 792 genes used for common inherited eye diseases on the Target_Eye_792_V2 chip.

No	Gene	OMIM	No	Gene	OMIM	No	Gene	OMIM	No	Gene	OMIM
1	*COLEC11*	612502	201	*FRAS1*	607830	401	*RGS9BP*	607814	601	*ARSE*	300180
2	*ATF6*	605537	202	*GRIP1*	601993	402	*SLC4A4*	603345	602	*EBP*	300205
3	*CNGA3*	600053	203	*FREM2*	608945	403	*TIMP3*	188826	603	*GNPAT*	602744
4	*CNGB3*	605080	204	*FRMD7*	300628	404	*ABCC6*	177850	604	*CNTN1*	600016
5	*GNAT2*	139340	205	*COL8A2*	120252	405	*RDH5*	601617	605	*RAD21*	606462
6	*PDE6H*	601190	206	*TCF4*	602272	406	*PAX2*	167409	606	*SMC1A*	300040
7	*MPZ*	159440	207	*ZEB1*	189909	407	*WDR19*	608151	607	*ALDH18A1*	138250
8	*HMCN1*	608548	208	*GALK1*	604313	408	*KCNV2*	607604	608	*ATP6V0A2*	611716
9	*TLR4*	603030	209	*GALE*	606953	409	*CACNA2D4*	608171	609	*EFEMP2*	604633
10	*CST3*	604312	210	*GALT*	606999	410	*CLCN7*	602727	610	*LTBP4*	604710
11	*CX3CR1*	601470	211	*ANTXR1*	606410	411	*HFE*	613609	611	*PYCR1*	179035
12	*CFI*	217030	212	*GBA*	606463	412	*ASRGL1*	609212	612	*PRX*	605725
13	*C2*	613927	213	*ROBO3*	608630	413	*CEP164*	614848	613	*DBH*	609312
14	*CFB*	138470	214	*ASB10*	615054	414	*GFAP*	137780	614	*ADAR*	146920
15	*C9*	120940	215	*GLB1*	611458	415	*ZFYVE26*	612012	615	*EMD*	300384
16	*FBLN5*	604580	216	*GM2A*	613109	416	*MIR204*	610942	616	*LMNA*	150330
17	*CFH*	134370	217	*HEXA*	606869	417	*RDH11*	607849	617	*SYNE2*	608442
18	*ERCC6*	609413	218	*MLPH*	606526	418	*TUB*	601197	618	*LAMA3*	600805
19	*HTRA1*	602194	219	*RAB27A*	603868	419	*ITM2B*	603904	619	*MYH11*	160745
20	*ARMS2*	611313	220	*OAT*	613349	420	*RBP4*	180250	620	*MYLK*	600922
21	*C3*	120700	221	*JAM3*	606871	421	*LAMA1*	150320	621	*PRKG1*	176894
22	*JAG1*	601920	222	*ACVRL1*	601284	422	*TREX1*	606609	622	*ASAH1*	613468
23	*NOTCH2*	600275	223	*ATL1*	606439	423	*PANK2*	606157	623	*PORCN*	300651
24	*COL4A4*	120131	224	*HPS1*	604982	424	*PRKCG*	176980	624	*FMR1*	309550
25	*COL4A5*	303630	225	*AP3B1*	603401	425	*TRNT1*	612907	625	*CTSA*	613111
26	*ALMS1*	606844	226	*HPS3*	606118	426	*ABHD12*	613599	626	*APC*	611731
27	*TP63*	603273	227	*HPS4*	606682	427	*MT-TP*	590075	627	*KIF1BP*	609367
28	*FGF10*	602115	228	*HPS5*	607521	428	*ANAPC1*	608473	628	*AGXT*	604285
29	*SLC2A10*	606145	229	*HPS6*	607522	429	*EMC1*	616846	629	*GRHPR*	604296
30	*APTX*	606350	230	*DTNBP1*	607145	430	*NEUROD1*	601724	630	*HOGA1*	613597
31	*SETX*	608465	231	*BLOC1S3*	609762	431	*OR2W3*	616729	631	*PTH*	168450
32	*FOXC1*	601090	232	*BLOC1S6*	604310	432	*SPP2*	602637	632	*STS*	300747
33	*PITX2*	601542	233	*CBS*	613381	433	*ADGRA3*	612303	633	*KDM6A*	300128
34	*ACTB*	102630	234	*IDUA*	252800	434	*AGBL5*	615900	634	*KMT2D*	602113
35	*ACTG1*	102560	235	*FTL*	134790	435	*ARL3*	604695	635	*ATP13A2*	610513
36	*GABRB1*	137190	236	*GCM2*	603716	436	*DHX38*	605584	636	*SGCB*	600900
37	*IFT27*	615870	237	*FAM126A*	610531	437	*EXOSC2*	602238	637	*SGCD*	601411
38	*BBS1*	209901	238	*GJB2*	121011	438	*HK1*	142600	638	*TTN*	188840
39	*BBS10*	610148	239	*IKBKG*	300248	439	*KIAA1549*	613344	639	*COX7B*	300885
40	*TRIM32*	602290	240	*ACO2*	100850	440	*KIZ*	615757	640	*CREBBP*	600140
41	*BBS12*	610683	241	*ADAMTSL4*	610113	441	*MVK*	251170	641	*TGFBR1*	190181
42	*MKS1*	609883	242	*RAX*	601881	442	*PRPF4*	607795	642	*TGFBR2*	190182
43	*WDPCP*	613580	243	*GDF6*	601147	443	*FLVCR1*	609144	643	*SMAD3*	603109
44	*SDCCAG8*	613524	244	*MFRP*	606227	444	*RP1*	603937	644	*TGFB2*	190220
45	*LZTFL1*	606568	245	*PRSS56*	613858	445	*IMPDH1*	146690	645	*TGFB3*	190230
46	*BBIP1*	613605	246	*GDF3*	606522	446	*PRPF31*	606419	646	*KCNH2*	152427
47	*BBS2*	606151	247	*CNNM4*	607805	447	*CRB1*	604210	647	*KCNJ2*	600681
48	*BBS4*	600374	248	*CSPP1*	611654	448	*PRPF8*	607300	648	*TSC1*	605284
49	*BBS5*	603650	249	*TCTN2*	613846	449	*TULP1*	602280	649	*TSC2*	191092
50	*MKKS*	604896	250	*B9D1*	614144	450	*CA4*	114760	650	*MAN2B1*	609458
51	*BBS7*	607590	251	*INPP5E*	613037	451	*PRPF3*	607301	651	*MANBA*	609489
52	*BBS9*	607968	252	*TTC21B*	612014	452	*ABCA4*	601691	652	*GNAS*	139320
53	*PTCH1*	601309	253	*KIF7*	611254	453	*RP2*	300757	653	*FLNA*	300017
54	*PTCH2*	603673	254	*TCTN1*	609863	454	*RPE65*	180069	654	*AUH*	600529
55	*NSD1*	606681	255	*TMEM237*	614423	455	*OFD1*	300170	655	*MMACHC*	609831
56	*PLA2G5*	601192	256	*CEP41*	610523	456	*EYS*	612424	656	*ASPM*	605481
57	*NOD2*	605956	257	*TMEM138*	614459	457	*CERKL*	608381	657	*CDK5RAP2*	608201
58	*FOXL2*	605597	258	*C5orf42*	614571	458	*NRL*	162080	658	*CEP135*	611423
59	*DRD5*	126453	259	*TCTN3*	613847	459	*FAM161A*	613596	659	*CEP152*	613529
60	*OPN1MW*	300822	260	*ZNF423*	604557	460	*RPGR*	312610	660	*ZNF335*	610827
61	*PHF6*	300414	261	*TMEM216*	613277	461	*FSCN2*	613596	661	*TACO1*	612958
62	*NR2F1*	132890	262	*TMEM231*	614949	462	*TOPORS*	609507	662	*DYSF*	603009
63	*RLBP1*	180090	263	*AHI1*	608894	463	*SNRNP200*	601664	663	*MCOLN1*	605248
64	*COL4A1*	120130	264	*ARL13B*	608922	464	*SEMA4A*	607292	664	*ARSB*	611542
65	*TFAP2A*	107580	265	*CC2D2A*	612013	465	*PRCD*	610598	665	*GALNS*	612222

66	*ZNF469*	612078	266	*SLC16A12*	611910	466	*NR2E3*	604485	666	*GNS*	607664
67	*PRDM5*	614161	267	*LRAT*	604863	467	*MERTK*	604705	667	*GUSB*	611499
68	*CYLD*	605018	268	*KIF21A*	608283	468	*RHO*	180380	668	*IDS*	300823
69	*GJA8*	600897	269	*COL18A1*	120328	469	*PDE6B*	180072	669	*NAGLU*	609701
70	*CRYBA1*	123610	270	*GALC*	606890	470	*PROM1*	604365	670	*MSH2*	609309
71	*PITX3*	602669	271	*C1QTNF5*	608752	471	*KLHL7*	611119	671	*ITGA7*	600536
72	*BFSP2*	603212	272	*CLUAP1*	616787	472	*PDE6A*	180071	672	*LAMA2*	156225
73	*GCNT2*	600429	273	*NPHP3*	608002	473	*RGR*	600342	673	*SEPN1*	606210
74	*GJA3*	121015	274	*PEX1*	602136	474	*CNGB1*	600724	674	*PLEC*	601282
75	*MIP*	154050	275	*GUCY2D*	600179	475	*IDH3B*	604526	675	*CHRNG*	100730
76	*CRYAB*	123590	276	*CEP290*	610142	476	*SAG*	181031	676	*DOK7*	610285
77	*CRYBB1*	600929	277	*RD3*	180040	477	*GUCA1B*	602275	677	*MSTN*	601788
78	*FYCO1*	607182	278	*RDH12*	608830	478	*CNGA1*	123825	678	*SCN4A*	603967
79	*LIM2*	154045	279	*KCNJ13*	603208	479	*TTC8*	608132	679	*DMPK*	605377
80	*CRYGC*	123680	280	*SPATA7*	609868	480	*C2orf71*	613425	680	*CNBP*	116955
81	*CRYGS*	123730	281	*AIPL1*	604392	481	*ARL6*	608845	681	*LMX1B*	602575
82	*MAF*	177075	282	*LCA5*	611408	482	*IMPG2*	607056	682	*NPC1*	607107
83	*CRYBB3*	123630	283	*RPGRIP1*	605446	483	*PDE6G*	180073	683	*TPM2*	190990
84	*CRYBA4*	123631	284	*CRX*	602225	484	*ZNF513*	613598	684	*TPM3*	191030
85	*CRYBB2*	123620	285	*NMNAT1*	608700	485	*DHDDS*	608172	685	*NEB*	256030
86	*VIM*	193060	286	*DTHD1*	616979	486	*PRPF6*	613979	686	*CFL2*	601443
87	*CHMP4B*	610897	287	*MT-ND1*	516000	487	*CLRN1*	606397	687	*SPINK5*	605010
88	*BFSP1*	603307	288	*COX10*	602125	488	*MAK*	154235	688	*PLA2G6*	603604
89	*TDRD7*	611258	289	*COX15*	603646	489	*C8orf37*	614477	689	*C19orf12*	614297
90	*AGK*	610345	290	*SURF1*	185620	490	*CDHR1*	609502	690	*COASY*	609855
91	*CRYGB*	123670	291	*OCRL*	300535	491	*RBP3*	180290	691	*NF2*	607379
92	*CRYGD*	123690	292	*CHST6*	605294	492	*NEK2*	604043	692	*MID1*	300552
93	*NHS*	300457	293	*ADIPOR1*	607945	493	*SLC7A14*	615720	693	*DDX59*	615464
94	*WFS1*	606201	294	*MT-TL1*	590050	494	*PRPH2*	179605	694	*TNFRSF11A*	603499
95	*CRYBA2*	600836	295	*SOD2*	147460	495	*IFT172*	607386	695	*OSTM1*	607649
96	*HSF4*	602438	296	*BEST1*	607854	496	*HGSNAT*	610453	696	*SNX10*	614780
97	*EPHA2*	176946	297	*IMPG1*	602870	497	*RP9*	607331	697	*TCIRG1*	604592
98	*CRYAA*	123580	298	*PRDM13*	616741	498	*GNPTG*	607838	698	*TNFSF11*	602642
99	*ABCA3*	601615	299	*CDH3*	114021	499	*ARL2BP*	615407	699	*SLC26A4*	605646
100	*IARS2*	612801	300	*IFT140*	614620	500	*RB1*	614041	700	*PEX10*	602859
101	*CCM2*	607929	301	*FREM1*	608944	501	*PLK4*	605031	701	*PEX11B*	603867
102	*ERCC2*	126340	302	*MAP2K1*	176872	502	*TINF2*	604319	702	*PEX12*	601758
103	*ERCC1*	126380	303	*FBN1*	134797	503	*KIAA0196*	610657	703	*PEX13*	601789
104	*CTC1*	613129	304	*SIL1*	608005	504	*RECQL4*	268400	704	*PEX14*	601791
105	*CHD7*	608892	305	*MAPKAPK3*	617111	505	*ESCO2*	609353	705	*PEX16*	603360
106	*SEMA3E*	608166	306	*KRT3*	148043	506	*UBIAD1*	611632	706	*PEX26*	608666
107	*LYST*	606897	307	*CHRDL1*	300350	507	*INVS*	243305	707	*PEX5*	600414
108	*PNPLA6*	603197	308	*TUBGCP4*	609610	508	*NPHP1*	607100	708	*PEX6*	601498
109	*KIF11*	148760	309	*TUBGCP6*	610053	509	*NPHP4*	607215	709	*STK11*	602216
110	*CHM*	303100	310	*VSX2*	142993	510	*IQCB1*	609237	710	*PGK1*	311800
111	*TMEM67*	609884	311	*SHH*	600725	511	*HESX1*	601802	711	*KIT*	164920
112	*ERCC8*	609412	312	*ABCB6*	605452	512	*ALDH3A2*	609523	712	*CHMP1A*	164010
113	*LONP1*	605490	313	*STRA6*	610745	513	*MTPAP*	613669	713	*EXOSC3*	606489
114	*PIGL*	605947	314	*ALDH1A3*	600463	514	*SYNE1*	608441	714	*RARS2*	611524
115	*OPN1LW*	300824	315	*DHODH*	126064	515	*ANO10*	613726	715	*TSEN2*	608753
116	*DRAM2*	613360	316	*RYR1*	180901	516	*EEF2*	130610	716	*TSEN34*	608754
117	*PCYT1A*	123695	317	*GFER*	600924	517	*ITPR1*	147265	717	*TSEN54*	608755
118	*UNC119*	604011	318	*ACTA2*	102620	518	*KCNC3*	176264	718	*VRK1*	602168
119	*C21orf2*	603191	319	*TRIM37*	605073	519	*KCND3*	605411	719	*UROD*	613521
120	*RAX2*	610362	320	*B4GAT1*	605517	520	*SPTBN2*	604985	720	*PEX2*	170993
121	*GUCA1A*	600364	321	*DAG1*	128239	521	*SYT14*	610949	721	*PEX7*	601757
122	*RAB28*	612994	322	*LARGE1*	603590	522	*TGM6*	613900	722	*PHYH*	602026
123	*TTLL5*	612268	323	*AGRN*	103320	523	*TTBK2*	611695	723	*AGPS*	603051
124	*POC1B*	614784	324	*CHAT*	118490	524	*WWOX*	605131	724	*TWIST1*	601622
125	*PDE6C*	600827	325	*COLQ*	603033	525	*ATXN10*	611150	725	*ACTA1*	102610
126	*PITPNM3*	608921	326	*P3H2*	610341	526	*ATXN7*	607640	726	*FHL1*	300163
127	*RIMS1*	606629	327	*LRPAP1*	104225	527	*ELOVL4*	605512	727	*TRPV4*	605427
128	*ADAM9*	602713	328	*PRIMPOL*	615421	528	*COL2A1*	120140	728	*HSPG2*	142461
129	*ACBD5*	616618	329	*ZNF644*	614159	529	*COL11A1*	120280	729	*NEU1*	608272
130	*MSMO1*	607545	330	*ADAMTS18*	607512	530	*COL11A2*	120290	730	*DHCR7*	602858
131	*CAV1*	601047	331	*CAPN5*	602537	531	*COL9A1*	120210	731	*NFIX*	164005
132	*SLC33A1*	603690	332	*LAMB2*	150325	532	*COL9A2*	120260	732	*AP4M1*	602296
133	*CTDP1*	604927	333	*PPT1*	600722	533	*TEAD1*	189967	733	*AP5Z1*	613653

134	*FBN2*	612570	334	*CTSD*	116840	534	*NAA10*	300013	734	*B4GALNT1*	601873
135	*POMT1*	607423	335	*GRN*	138945	535	*VAX1*	604294	735	*CYP2U1*	610670
136	*TMEM5*	605862	336	*CTSF*	603539	536	*RARB*	180220	736	*CYP7B1*	603711
137	*B3GALNT2*	610194	337	*TPP1*	607998	537	*HMGB3*	300193	737	*DDHD2*	615003
138	*POMK*	615247	338	*CLN3*	607042	538	*MAB21L2*	604357	738	*ERLIN2*	611605
139	*GMPPB*	615320	339	*DNAJC5*	611203	539	*BCOR*	300485	739	*FA2H*	611026
140	*POMT2*	607439	340	*CLN6*	608102	540	*SOX2*	184429	740	*GBA2*	609471
141	*POMGNT1*	606822	341	*CLN5*	608102	541	*OTX2*	600037	741	*HSPD1*	118190
142	*FKTN*	607440	342	*MFSD8*	611124	542	*BMP4*	112262	742	*KIF1A*	601255
143	*FKRP*	606596	343	*CLN8*	607837	543	*HCCS*	300056	743	*KIF5A*	602821
144	*ISPD*	614631	344	*MT-ATP6*	516060	544	*TTR*	105210	744	*MARS2*	609728
145	*POMGNT2*	614828	345	*ABHD5*	275630	545	*TCOF1*	606847	745	*NIPA1*	608145
146	*GNAT1*	139330	346	*NPC2*	601015	546	*OPN1SW*	613522	746	*REEP1*	609139
147	*GRK1*	180381	347	*SMPD1*	607608	547	*TUBB3*	602661	747	*RTN2*	603183
148	*CACNA1F*	300110	348	*PTPN11*	176876	548	*ADGRV1*	602851	748	*SPG11*	610844
149	*NYX*	300278	349	*KRAS*	190070	549	*WHRN*	607928	749	*SPG7*	602783
150	*TRPM1*	603576	350	*SOS1*	182530	550	*CEP250*	609689	750	*ZFYVE27*	610243
151	*SLC24A1*	603617	351	*RAF1*	164760	551	*MYO7A*	276903	751	*MAPT*	157140
152	*GRM6*	604096	352	*NRAS*	164790	552	*USH1C*	605242	752	*SALL1*	602218
153	*CABP4*	608965	353	*BRAF*	164757	553	*CDH23*	605516	753	*POLR1C*	610060
154	*GPR179*	614515	354	*NDP*	300658	554	*PCDH15*	605514	754	*BAP1*	603089
155	*LRIT3*	615004	355	*RP1L1*	608581	555	*USH1G*	607696	755	*IRF6*	607199
156	*GNB3*	139130	356	*IGBP1*	300139	556	*USH2A*	608400	756	*TBX1*	602054
157	*DCN*	125255	357	*HMX1*	142992	557	*PDZD7*	612971	757	*EPG5*	615068
158	*PLG*	173350	358	*OCA2*	611409	558	*HARS*	142810	758	*ERCC3*	133510
159	*KERA*	603288	359	*TYRP1*	115501	559	*CIB2*	605564	759	*XPA*	611153
160	*TGFBI*	601692	360	*SLC45A2*	606574	560	*MT-TS2*	590085	760	*XPC*	613208
161	*TACSTD2*	137290	361	*SLC24A5*	609802	561	*VCAN*	118661	761	*BLM*	210900
162	*KRT12*	601687	362	*C10orf11*	614537	562	*VHL*	608537	762	*GRHPR*	604296
163	*VSX1*	605020	363	*GJA1*	121014	563	*SMOC1*	608488	763	*CEP135*	611423
164	*SLC4A11*	610206	364	*PABPN1*	602279	564	*TYR*	606933	764	*COASY*	609855
165	*PIKFYVE*	609414	365	*KCTD7*	611725	565	*PAX3*	606597	765	*STK11*	602216
166	*PXDN*	605158	366	*ATP1A3*	182350	566	*MITF*	156845	766	*VRK1*	602168
167	*CYP4V2*	608614	367	*RTN4IP1*	610502	567	*SNAI2*	602150	767	*CYP2U1*	610670
168	*SLITRK6*	609681	368	*UCHL1*	191342	568	*EDNRB*	131244	768	*NIPA1*	608145
169	*TIMM8A*	300356	369	*AFG3L2*	604581	569	*EDN3*	131242	769	*CAV3*	601253
170	*EP300*	602700	370	*C12orf65*	613541	570	*SOX10*	602229	770	*LAMB3*	150310
171	*FUCA1*	612280	371	*PRPS1*	311850	571	*RAB3GAP1*	602536	771	*LAMC2*	150292
172	*ITGB3*	173470	372	*SLC25A46*	610826	572	*RAB3GAP2*	609275	772	*ITGA2B*	607759
173	*ROM1*	180721	373	*OPA1*	605290	573	*RAB18*	602207	773	*ANO5*	608662
174	*EFEMP1*	601548	374	*OPA3*	606580	574	*TBC1D20*	611663	774	*GDF2*	605120
175	*CHN1*	118423	375	*TMEM126A*	612988	575	*ADAMTS10*	608990	775	*SHOX*	312865
176	*SALL4*	607343	376	*MFN2*	608507	576	*ADAMTS17*	607511	776	*SGCG*	608896
177	*ABCC2*	601107	377	*SIX6*	606326	577	*WRN*	604611	777	*SGCA*	600119
178	*ACD*	609377	378	*NBAS*	608025	578	*TLR2*	603028	778	*TCAP*	604488
179	*NHP2*	606470	379	*PAX6*	607108	579	*ATP7B*	606882	779	*KCNQ1*	607542
180	*NOP10*	606471	380	*GP1BA*	606672	580	*NIPBL*	608667	780	*CUBN*	602997
181	*TERT*	187270	381	*DMD*	300377	581	*HDAC8*	300269	781	*CENPJ*	609279
182	*WRAP53*	612661	382	*CTNNA1*	116805	582	*CISD2*	611507	782	*MCPH1*	607117
183	*EDARADD*	606603	383	*B3GLCT*	610308	583	*RS1*	300839	783	*STIL*	181590
184	*PKP1*	601975	384	*PHOX2A*	602753	584	*GPR143*	300808	784	*HYAL1*	607071
185	*COL3A1*	120180	385	*MT-TH*	590040	585	*DNAJC19*	608977	785	*CAPN3*	114240
186	*PLOD1*	153454	386	*TUBA8*	605742	586	*SF3B4*	605593	786	*MYH7*	609928
187	*TNXB*	600985	387	*MYOC*	601652	587	*PIK3R1*	171833	787	*MYOT*	604103
188	*LOXL1*	153456	388	*OPTN*	602432	588	*HGD*	607474	788	*TNNT1*	191041
189	*ZNF408*	616454	389	*WDR36*	609669	589	*KCNJ10*	602208	789	*SEPSECS*	613009
190	*MC1R*	155555	390	*NTF4*	162662	590	*MRE11A*	600814	790	*ATP7A*	300011
191	*IKBKAP*	603722	391	*CYP1B1*	601771	591	*ATM*	607585	791	*HPD*	609695
192	*RCBTB1*	607867	392	*LTBP2*	602091	592	*ATP2C1*	604384	792	*TAT*	*613018*
193	*FZD4*	604579	393	*POLG*	174763	593	*COL6A3*	120250			
194	*LRP5*	603506	394	*RNASEH1*	604123	594	*COL6A2*	120240			
195	*TSPAN12*	613138	395	*SLC25A4*	103220	595	*BLM*	210900			
196	*FGFR1*	136350	396	*C10orf2*	606075	596	*MTM1*	300415			
197	*FGFR2*	176943	397	*POLG2*	604983	597	*CYP27A1*	606530			
198	*FGFR3*	134934	398	*RRM2B*	604712	598	*TFAP2B*	601601			
199	*LCAT*	606967	399	*DNA2*	601810	599	*DNM2*	602378			
200	*FOXE3*	601094	400	*RGS9*	604067	600	*PDK3*	300906			
